# The Immunotherapeutic Role of Type I and III Interferons in Melanoma and Non-Melanoma Skin Cancers

**DOI:** 10.3390/life13061310

**Published:** 2023-06-01

**Authors:** Sydney A. Weir, Kailash KC, Shoaib Shoaib, Nabiha Yusuf

**Affiliations:** 1 Heersink School of Medicine, University of Alabama at Birmingham, Birmingham, AL 35294, USA; sydneyaw@uab.edu; 2 School of Medicine, Alabama College of Osteopathic Medicine, Dothan, AL 36303, USA; 3 Department of Biochemistry, Faculty of Medicine, Aligarh Muslim University, Aligarh 202001, UP, India; 4 Department of Dermatology, Heersink School of Medicine, University of Alabama at Birmingham, Birmingham, AL 35294, USA

**Keywords:** interferon, skin cancer, cancer therapeutics, melanoma, squamous cell carcinoma, basal cell carcinoma, immunotherapy

## Abstract

Interferons (IFNs) have demonstrated therapeutic potential in various skin cancers, specifically squamous cell carcinoma (SCC), basal cell carcinoma (BCC), and melanoma. The precise mechanism through which type I IFNs exert their antitumor effects in skin cancers is still being studied. However, intralesional type I IFN can be used as an alternative to surgery for select patient populations, and high-dose systemic IFN therapy has been shown to be promising in patients with operable high-risk or metastatic melanoma. Despite the therapeutic potential of IFNs in skin cancer treatment, the toxicity profile often prevents the completion of treatment and further expansion of its clinical application. Type I and III IFNs use the same Janus Kinases (JAKs) for signal transduction, which are pathways initiated at a cell surface receptor that mediates the activation of target genes in the nucleus, based on this shared signaling pathway. Due to selective tumor targeting and the ability to generate both innate and adaptive immune responses, we concluded that type III IFNs have minimal side effects compared with established treatments due to selective tumor targeting. While IFN-λ, a type III IFN, shows therapeutic potential as stand-alone or in combination with another IFN, further studies need to be conducted to explore the therapeutic potential of IFN-λ in skin cancer and the underlying physiological roles and mechanisms of action. In this review, we evaluate whether treatment of skin cancer with type III IFN will have minimal side effects compared with established treatments.

## 1. Introduction

Interferons (IFNs) are a group of cytokines, initially named for their ability to interfere with influenza virus replication, and are classified into three families: type I, type II, and type III [1]. Both type I and type III IFNs signal through heterodimeric receptors, induced by similar pathogen-sensing pathways and exhibit substantial overlap in transcriptional responses and downstream signaling pathways [2]. The critical difference between type I and III IFN signaling lies in their cell-type specific reactions resulting from the localization of their receptors. While the IFN-α receptor 1 (IFNARs) are ubiquitously expressed, the IFN-λ receptor 1 (IFNLR1) chain is primarily expressed in epithelial cells [2].

Numerous studies have established the therapeutic efficacy of type I IFNs as an anticancer treatment across various malignancies. The role of type I IFNs in cancer has been investigated for over 50 years, with initial studies showing antitumor effects in mouse models and subsequent clinical trials demonstrating efficacy in hematological malignancies and solid tumors [3], including breast and renal malignancies and melanoma. The precise mechanism through which type I IFNs exert their antitumor effects in melanoma and non-melanoma skin cancers is still being studied [4]. However, recent research has highlighted the importance of endogenous IFN-I in controlling tumor growth and response to antitumor therapies [3]. Despite its therapeutic potential in cancer treatment, the toxicity profile of type I IFNs often prevents the completion of treatment and further expansion of its clinical application [3].

Based on the knowledge of the shared signaling pathways between type I and type III IFNs and their restrictive effects in relation to various cell types, in this review, we evaluate the hypothesis that treatment of skin cancer with type III IFN will have minimal side effects compared with established treatments due to selective tumor targeting and the ability to generate both innate and adaptive immune responses.

## 2. Materials and Methods

This literature review was conducted on PubMed and included the following search terms: interferon(s), skin cancer, squamous cell carcinoma, basal cell carcinoma, melanoma, IFN, and cancer therapeutics.

## 3. Results

### 3.1. Type I and Type III Interferons and Their Biological Actions

Interferons (IFNs) are a broad class of cytokines, initially named for their ability to interfere with influenza virus replication [5,6]. IFNs represent the first-line defense against a broad range of viruses and pathogens by stimulating the body’s innate immune response [6]. IFNs regulate over 200 gene products at the transcriptional level [7]. Based on their sequence homology and signaling properties, IFNs are classified into three families: type I, type II, and type III. For all classes, the formation of the IFN receptor complex activates Janus Kinases (JAKs) that result in IFN-mediated intracellular signaling cascades [8]. Specifically, type I and type III IFN use the same JAKs for signal transduction discussed in subsequent sections. 

**Figure 1:**
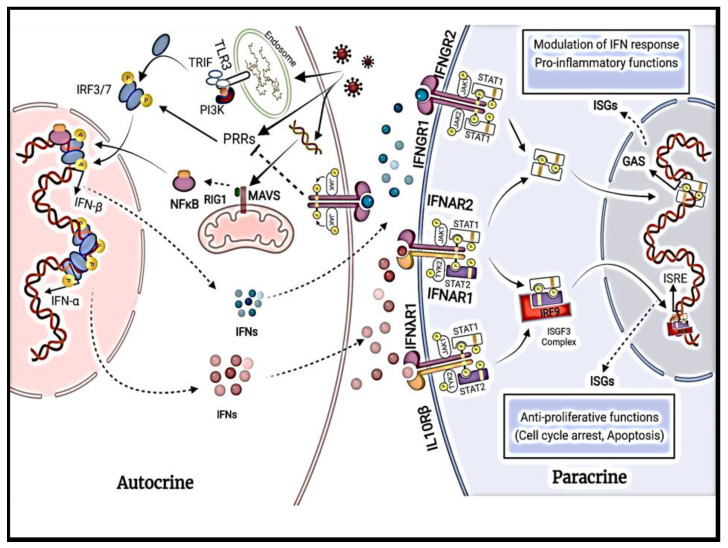
Detailed illustration of autocrine and paracrine signaling effects of IFN-α and IFN-β.

Type II IFNs, consisting of IFN-γ, have low sequence similarity with type I and type III IFNs [9,10]. Type II IFNs act directly and indirectly on tumor cells, through increasing the expression of MHC class I molecules on the surface of tumor cells, increasing antigenicity, and inhibiting tumor growth [11]. The role of IFN-γ in regulating immune status and antitumor immunity is controversial, as it can stimulate host immune response and improve the efficiency of various cancer therapies but can also reduce immune response and stimulate tumor progression and metastasis [11]. Several critical determinants for the anti- and protumorigenic effects of type II IFNs and their role in cancer patients need to be fully understood to optimize their potential in cancer treatment [11]. While type II IFNs are produced by immune cells such as activated T cells and natural killer (NK) cells upon induction by other cytokines [9,12], type I and type III IFNs are produced by both immune and tissue-specific cells and will be the focus of this paper. Although type I and type III IFNs were initially recognized for their antiviral activity, they also exert immunomodulatory responses to tumors, autoimmune diseases, and microbial infections [2]. The autocrine and paracrine effects of type I and III interferon signaling is summarized in Figure 1.

#### 3.1.1. Type I Interferons

Type I IFNs are the most prominent family of cytokines in humans consisting of 13 subtypes of IFN-α and 1 subtype each of IFN-β, IFN-ε, IFN-κ, and IFN-ω [10]. The IFN-α and IFN-β subtypes are the best-characterized and most widely expressed genes; the other subtypes remain poorly understood due to low expression levels and overlapping functions [13]. While all subtypes exhibit limited structural similarity, they signal through the same heterodimeric receptor with one chain of IFN-α receptor 1 (IFNAR1) and one chain of IFN-α receptor 2 (IFNAR2) [14,15]. IFNARs are ubiquitously expressed [16]. IFNAR1 and IFNAR2 signal through JAK, tyrosine kinase 2 (TYK2), and JAK1, respectively; the activation of JAKs causes the tyrosine phosphorylation of signal transducer and the activator of transcription 1 (STAT1) and STAT2 (shown in Figure 1). This cascade, in turn, leads to the formation of a trimeric complex called IFN-stimulated gene factor 3 (ISGF3) [13]. ISGF3 initiates gene transcription by translocating to the nucleus and binding to IFN-stimulated response elements (ISREs) in the gene promoters of IFN-inducible genes [13]. Furthermore, IFNAR activation also initiates STAT1 homodimers that bind and stimulate gamma-activated sequence (GAS) motifs, which leads to the induction of gene transcription [13].

#### 3.1.2. Type III Interferons

Type III IFNs consist of four subtypes of IFN-λ: IFN-λ1 (IL-29), IFN-λ2 (IL-28A), IFN-λ3 (IL-28B), and IFN-λ4 [17]. They signal through heterodimeric receptors with one chain of IFN-λ receptor 1 (IFNLR1) and one chain of interleukin-10 receptor 2 (IL-10R2) [18,19,20]. The IL-10-R2 chain is ubiquitously expressed, while the IFNLR1 receptor is only expressed in cells of epithelial origin, plasmacytoid dendritic cells, neutrophils, NK cells, and B cells [17]. Structurally, type III IFNs are related to type I IFNs and the IL-10 family. Like type I IFNs, the type III IFN class signals through the activation of JAK1 and TYK2 [13]. The activation of JAKs leads to the phosphorylation of the receptor complex and the subsequent recruitment of STAT1 and STAT2 to form the ISG3 transcription complex [13].

Regarding IFN gene response, IFN-λ-induced IFN-stimulated genes (ISGs) are practically identical to type I IFN-induced ISGs described previously. The key difference between the two is that type III IFN has a smaller spectrum of ISG activation. Furthermore, type III IFNs do not induce any unique ISGs, and compared with type I IFNs, type IIIs have a lower magnitude but longer-lasting ISG induction [17]. Despite the substantial overlap in their signaling cascade, growing evidence suggests spatial and kinetic differences in type I and type III IFN responses, thus leading to unique context-specific functional responses, reviewed in detail elsewhere [2].

### 3.2. Immunotherapy with Type I and III Interferons

Cancer immunotherapy has rapidly advanced in recent years [21]. The main goal of cancer immunotherapy is to activate passive or active immunity to target tumors and combat growing malignancies [22]. Biologic agents such as antibodies, checkpoint inhibitors, and cytokines are a few of the many that comprise cancer immunotherapy [23]. Our understanding of basic tumor immunology has primarily driven the development of novel cancer therapeutics [24]. IFNs regulate each step of the cancer immunity cycle, leaving several potential steps to target in the development of therapeutics. The cancer immunity cycle refers to the cyclic process during which neoantigens produced by cancer cells are released after cell death, captured, and then presented to T cells by antigen-presenting cells, activating effector T cells [25]. Effector T cells then traffic and infiltrate tumor tissue, where they kill cancer cells with the same tumor antigens, causing more antigen release [22]. Several studies have revealed that IFN signaling plays a critical role in the success of cancer therapy strategies. However, IFNs can also support cancer progression and mediate cancer immune escape [25,26]. We will discuss the roles of type I and III interferons in a variety of cancer therapies. 

#### 3.2.1. Type I Interferons

Type I interferons modulate translational and post-translational modifications of proteins and protein degradation, induce apoptosis, and can cause cell cycle arrest [27]. Recently, the focus of cancer research has shifted from drug or gene therapy to cell processes such as protein synthesis as an antitumor strategy. Regarding translational and post-translational modifications of proteins, two important targets of type I IFNs are the protein kinase-dependent on double-stranded RNA (PKR) gene and the eukaryotic initiation factor-5A of protein synthesis (eIF-5A) [27]. Human PKR is a serine–threonine kinase involved in promoting cell death mediated by double-stranded RNA. PKR also regulates several transcription factors, including nuclear factor (NF)-kB, interferon regulatory factor-1 (IRF-1), p53, STAT1, and NF-90 [27]. PKR plays a vital role in controlling cellular processes such as cell growth, differentiation, and antitumor activity by regulating critical transcription factors. PKR’s antitumor activity involves inducing cell cycle arrest and apoptosis in cancer cells [27].

Another critical target of type I IFNs is eIF-5A, a protein that belongs to a family of translational factors involved in regulating cell growth and differentiation. IFN-α induces cell growth inhibition, apoptosis, and a reduction in the activity of eIF-5A in human epidermoid cancer cells, highlighting the role of eIF-5A in IFN-α-induced apoptosis [27].

Studies have shown that the type I IFN (IFN-α/β) exhibits antitumor activity through mechanisms depicted in Figure 1. Experiments by Yaar et al. [28] showed that cultures of human keratinocytes supplemented with 2500 units/mL of either IFN-α or IFN-β demonstrated mean growth inhibition of 70% in seven days compared with control cultures [4]. IFN-α and IFN-β also promoted keratinocyte terminal differentiation, as demonstrated by increased cornified envelope formation and cell shedding in IFN-treated cultures compared with control cultures. These effects on growth and terminal differentiation were reversible upon the withdrawal of IFN from the medium [4,28]. Furthermore, mouse IFN-α/β levels have been shown to inhibit experimental wound healing in mice by inhibiting the proliferation of many different cell types, including endothelial, epidermal, and connective tissue cells [4,29]. Studies have shown that IFN-α upregulates the expression of class I, but not class II, major histocompatibility complex (MHC) antigens in cultured human keratinocytes. IFN-α and IFN-β induce increased expression of class I MHC antigens in cultured human melanocytes, with IFN-β having a more significant effect than IFN-α [4,30,31]. As shown in Figure 1, IFN-α and IFN-β both exhibit a vast therapeutic potential, specifically in skin cancers. IFN-α signaling also leads to the co-expression of CD95L and CD95 in nodular BCCs, leading to cell death via suicide and fratricide [4,32].

Further studies have shown that the in vitro and in vivo inactivation of type I IFN signaling by using shIFNAR1 cells and IFNAR1-null mice, respectively, overcome oncogene-induced senescence, a tumor-suppressive signal that protects DNA-damaged cells from the onset of melanoma. Type I IFNs are produced after DNA damage and contribute to the senescence of cancer cells. In melanoma, BRAF activation and mutations in phosphoinositide 3-kinase (PI3K) can downregulate IFNAR1, which inhibits the tumor-suppressive role of IFN signaling [33].

In a recent mini-review, the role of retinoic acid-inducible gene I (RIG-I)-like receptors (RLRs) and type I IFNs in the treatment of glioblastoma (GB), a common aggressive primary adult brain cancer, was assessed [34]. RLRs, part of the host innate immunity in the family of pattern recognition receptors, are vital sensors of viral and host-derived RNAs. Furthermore, RLRs are essential mediators in the activation of the innate immune system through type I IFNs. Once RLRs bind to immunostimulatory RNAs, they undergo conformational changes to become activated. The activation of RIG-I and melanoma differentiation-associated factor 5 (MDA-5), a cytoplasmic viral RNA detector, induces the exposition of their caspase activation and recruitment domains to interact with mitochondrial antiviral signaling protein (MAVS), an adaptor protein that initiates the RLR cascade. Subsequently, IRFs 1, 3, and 7 are activated with NF-κB, which leads to the expression of type I IFN and other genes, such as ISGs; the cumulative effect of these cellular events is an antitumor immune response and cancer cell apoptosis. In vitro, studies have provided evidence on the potential benefit of RLR agonists on inhibiting GB tumor growth and have shown that treatment with IFN-β can augment treatment response. Further studies have investigated RLR agonists with radiotherapy; ionizing radiotherapy treatment activates type I IFN production, subsequently promoting tumor cytotoxicity and immune response activation. Based on the role of RLR in promoting type I IFN response and inducing cancer cell apoptosis, RLR agonists are currently under investigation in clinical trials, in combination with radiation, in adults with GB [34].

Another example of type I IFN antitumor properties in melanoma is that the in vivo growth of melanoma cells is dependent on prostaglandin E2 (PGE2) production from the cyclooxygenase (COX) pathway. An excess of PGE2 limits type I IFN-secreting immune cells, allowing the tumor cell to proliferate unchecked. In contrast, studies on type III IFN (IFN-λ) and its true potential as anticancer therapeutics are limited. However, a recent study found that IFN-λ directly upregulates antiproliferative and proapoptotic regulator molecules in tumor cells [25]. Additionally, as discussed previously, IFN-λ induces chemokines that recruit CD4+ T cells into the tumor microenvironment [33,35].

In 2021, Zhang et al. extensively reviewed how IFNs regulate each step of the cancer immunity cycle and how IFNs at each step may be a target for treatment [25,33]. Our understanding of the mechanisms of IFNs, coupled with how they regulate each step of the cancer immunity cycle, provides insight into the therapeutic potential of IFNs. 

Furthermore, IFN-α enhances cell-mediated cytotoxicity, decreases T helper 2 (Th2) cell production by tumor cells, and inhibits malignant T-cell proliferation. Through these mechanisms, IFN-α has been used to treat cutaneous T-cell lymphoma (CTCL) and augment therapy with psoralen plus ultraviolet A (UVA) light phototherapy [36]. One study found the possible efficacy of IFN-α when combined with oral retinoids, photopheresis, total skin electron beam therapy (TSEBT), and narrowband UVB light phototherapy [36]. Specifically, in the treatment of CTCL, several other targets stimulate IFN production and augment cancer therapeutics. Studies have shown that targeting interleukin-12 (IL-12), Toll-like receptors (TLRs), programmed cell death protein 1 (PD1), and CD47 promotes the production of IFNs and other cytokines that contribute to a robust antitumor immune response. 

While the various mechanisms in which type I IFNs have antitumor activity have been highlighted, there is growing evidence that the deficiency of IFN signaling is one of the most significant reasons for immune dysfunction and the resistance or failure of traditional cancer treatments. Several studies have found that impaired ISG expression in lymphocytes of certain malignancies, including melanoma, breast, and gastrointestinal cancers, led to defects in IFN signaling in lymphocytes, suggesting a common cancer-associated mechanism of immune dysfunction [25]. Furthermore, while the positive roles of IFNs have been highlighted, research has also shown that IFNs induce therapy resistance by mediating cancer immune escape in tumor microenvironments. One example is that the sustained activation of type I IFN induces the upregulation of programmed cell death ligand 1 (PD-L1) in both tumor and dendritic cells and enhances the expression of nitric oxide synthase 2 (NOS2), leading to eventual resistance to PD-1 blockade. Research has also suggested that type I IFN also induces radiation resistance by enhancing the recruitment of immunosuppressive myeloid cells via the C–C motif chemokine receptor 2 (CCR2) pathway [25].

The data on IFN as a stand-alone therapy or in combination with other cancer therapeutics have been growing. The research community has also highlighted that a substantial number of patients fail to respond to these immunotherapies [37]. Amouzegar et al. investigated a target that could elicit or augment antitumor immune responses called a stimulator of interferon genes (STING). STING is an endoplasmic protein that induces the production of proinflammatory cytokines, including type I IFNs. STING agonists delivered via antibody–drug conjugates have shown promising preclinical results. In vitro assays, with STING agonist antibody–drug conjugates in two murine tumor models, showed significant inhibition in tumor growth when compared to a control. Furthermore, in an intratumoral checkpoint refractory B16-F10 melanoma murine model, STING treatment showed substantial induction of PD-L1 expression [37].

#### 3.2.2. The Clinical Consideration of Type I Interferons 

IFN-α was approved by the United States Food and Drug Administration to be used as the standard of care in advanced melanoma; however, its’ further clinical application is hampered by the high cost of drug administration, limited efficacy, and adverse constitutional events. Serious side effects of the systemic administration of IFN-α are associated with higher doses. A few of the severe adverse effects include cytopenia (e.g., neutropenia, lymphopenia, and thrombocytopenia), gastrointestinal dysfunction (e.g., nausea, vomiting, and anorexia), and nervous system effects (e.g., fatigue, depression, and suicidal ideation) [35,38].

The development of cell-based therapy has primarily overcome the serious side effects related to IFN-α. Cell-based therapy involves modifying cells, such as mesenchymal stem cells (MSCs), to express IFN-α and convey it to the tumor site. MSCs are great options for cell-based therapy because they are easily isolated, expand ex vivo, and transduce with viral or non-viral vector encoding IFN-α. MSC therapy is currently being investigated to determine if adjusting the number of MSCs and the quantity of IFN-α secreted could enhance the involvement of the immune system in fighting cancer [33].

#### 3.2.3. Type III Interferons

Type III interferons have potent antiviral properties, play a role in chronic inflammation, and have antitumor activity [17]. One of the main functions of type III IFNs is to inhibit viral infections. As a key defense mechanism in a human’s innate immune system, type III IFNs exhibit antiviral properties by inhibiting several steps of the viral replication cycle. Furthermore, type III IFNs strengthen epithelial barriers in the gastrointestinal and respiratory tracts and the central nervous system. The strengthening of these epithelial barriers protects against viral invasion and systemic infection in a manner that is independent of the JAK/STAT signaling pathway [17]. Type III IFNs also enhance the production of proinflammatory cytokines and chemokines, leading to the recruitment of immune cells to sites of inflammation. Lastly, type III interferons have significant antiproliferative and proapoptotic effects on tumor cells. The induction of chemokines such as CXCL-9 or CXCL-10 by IFN-λ recruits CD4+ T cells into the tumor environment, and antitumor responses of CD8+ T cells and natural killer (NK) cells are enhanced in various types of tumors. 

Despite limited research, studies have shown that melanoma cells were among the few that responded well to IFN-λ [38]. In melanoma, the tumor-targeting mechanism of type III is comparatively similar to type I, including antiangiogenesis, cell cycle arrest, antimitotic, and apoptosis [38]. Studies have also shown that type III IFN plays an immunomodulatory role in melanoma by creating a microenvironment including T cells and NK cells [38]. A clinical study investigating IFN-λ as a treatment for chronic hepatitis C indicated that, unlike IFN-α and IFN-β, IFN-λ might decrease tumor burden while increasing tumor response without any significant myelosuppression [38,39,40]. Furthermore, the patients receiving IFN-λ did not develop neutropenia and thrombocytopenia, suggesting a therapeutic potential with lesser side effects than IFN-α and IFN-β. In addition, more T cells may reach the tumor microenvironment [38,39,40].

**Figure 2:**
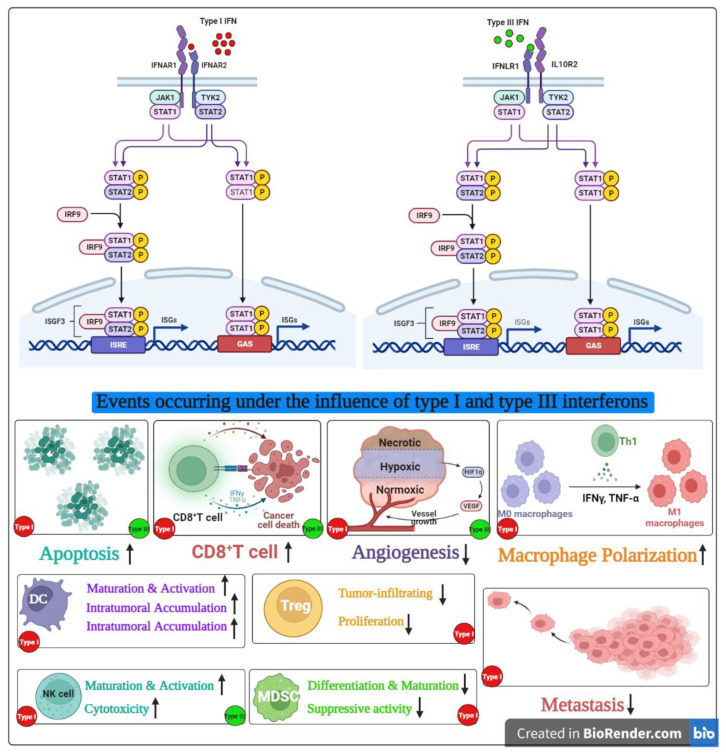
Overlapping antitumor functions of type I and III interferons.

In a study performed in B16F10 melanoma cells, IFN-λ exhibited its antitumor effect in a dose-dependent manner. In a poorly immunogenic tumor such as melanoma, high levels of constitutive MHC class I antigen expression via IFN-λ induction may render the cells more immunogenic and promote adaptive antitumor immune responses. However, the same study also suggested that upregulation of MHC class I antigen expression may not be sufficient to increase the immunogenicity and promote adaptive antitumor immune response because tumor cells with IFN-λ did not stimulate a memory response [41]. In addition, the study found that the significant players in antitumor immunity, such as primary lymphocytes and macrophages, were not responsive to IFN-λ, suggesting that while the immune cells are not the primary target, the host defense mechanisms played a key role [41]. The overlapping antitumor mechanisms of type I and III interferons are summarized in Figure 2.

In normal cells, both type I and III IFNs function as immunomodulators. While type I IFNs elevate both innate and adaptive immune responses, type III IFNs seem to support cell-mediated immunity by upregulating the class I expression [35].

IFN-λ has also been suggested to contribute to antiangiogenic mechanisms. Mainly, IFN-λ inhibits the stimulation of angiogenesis by tumor or stroma, which is essential for tumor survival and proliferation in vivo [41,42]. However, the mechanism of action of how IFN-λ contributes to antitumor effects and how these mechanisms compare with that exhibited by type I IFNs warrant further studies [41,43]. Table 1 summarizes the uses of type I and type III IFNs in squamous cell carcinoma, basal cell carcinoma, and melanoma. 

## 4. Discussion

Various studies have demonstrated the therapeutic potential of IFNs as anticancer drugs, specifically in treating squamous cell carcinoma, basal cell carcinoma, and melanoma [4]. Although approved for the treatment of several diseases, some of which include Kaposi’s sarcoma, melanoma, chronic myeloid leukemia, and chronic granulomatous disease, type I and type II IFNs have severe adverse effects largely because of the ubiquitous expression of their receptors [51]. Although IFN-α is currently the most widely used IFN in clinical settings [33], type III IFNs function similarly to type I IFNs as antiviral, immunomodulatory, and antiproliferative agents. However, unlike type I IFN receptors that are widely expressed, type III IFN receptors exhibit restricted patterns of expression [33]. Thus, through selective tumor targeting, IFNLR expression on fewer cells, and the ability to generate innate and adaptive immune responses, IFN-λ offers unique advantages of minimal side effects [41].

The therapeutic use of interferons in non-melanoma skin cancer has shown to be promising. Studies have shown that intra-lesional IFN-α is an effective treatment for superficial BCC and cutaneous SCC [44,45,46]. A more recent study concluded peri- and intra-lesional combination of IFN-α and IFN-γ was safe and effective for advanced and recurrent BCC and cutaneous SCC in older patients. Side effects included influenza-like symptoms, which were mild and well tolerated [46]. While research on the systemic use of IFN-λ is growing, to the best of our knowledge, there are currently no studies investigating intra-lesional IFN-λ in the treatment of non-melanoma skin cancers. 

As far as melanoma is concerned, IFN-λ has been shown to be beneficial as a second-line therapy when combined with low-dose IFN-α or traditional anticancer agents. One study showed that IFN-λ, when used with a subtherapeutic dose of IFN-α, improved the therapy with less toxic side effects among cancer patients [38,49]. However, new studies have shown that IFN-λ can also promote oncogenesis, which must be considered when utilizing the cytokine as a drug [38]. Additionally, inherent or developed insensitivity to IFN therapy is another concern to consider, especially in more aggressive tumors [50]. More research is needed to evaluate whether type III IFNs would be a safe and efficacious alternative for IFN-α therapy. While type III IFN cancer therapy may be promising, it appears developing research continues to focus on type I and type II IFN applications in cancer [3,50,52,53], further highlighting the need to investigate the use of type III IFNs. 

Through this extensive literature review, we concluded that type III IFNs have minimal side effects, compared with established treatments, due to selective tumor targeting. Together, while IFN-λ shows therapeutic potential as stand-alone or in combination with another IFN, further studies need to be conducted to explore the therapeutic potential of IFN-λ in skin cancer and the underlying physiological roles and mechanisms of action.

## Figures and Tables

**Table 1:** Comparison of type I and type III IFN therapies in non-melanoma and melanoma skin cancers.

	Type I IFN	Type III IFN
**Squamous Cell Carcinoma**	*Route of administration:* Peri- or Intra-lesional (IFN-α as a stand-alone therapy or in combination with IFN-γ) [44,45,46] *Current Recommendations:* Intra-lesional Type I IFN therapy is seldom used for BCC or SCC due to limited evidence on the long-term effectiveness of these treatments, typically reserved for patients who cannot undergo surgery [47] *Therapeutic Concerns:* side effects (influenza-like symptoms, which were mild and well tolerated) [46]	Currently, no studies investigating intra-lesional IFN-λ in the treatment of non-melanoma skin cancers
**Basal Cell Carcinoma**		
**Melanoma**	*Route of administration:* Systemic *Current recommendations:* IFN-α standard of care in advanced melanoma [41] *Anticancer mechanisms of action (also depicted in Figure 1):* antiangiogenesis, cell cycle arrest, antimitotic, and proapoptotic [38] *Therapeutic Concerns:* Adverse effects include cytopenia (e.g., neutropenia, lymphopenia, and thrombocytopenia), gastrointestinal dysfunction (e.g., nausea, vomiting, and anorexia), and nervous system effects (e.g., fatigue, depression, and suicidal ideation) [35,38]	*Route of administration:* Systemic and dose-dependent antitumor effect [42] *Current recommendations:* IFN-λ beneficial as second-line therapy when combined with low-dose IFN-α or traditional anticancer agents [38,48,49] *Anticancer mechanisms of action:* antiangiogenesis, cell cycle arrest, antimitotic, proapoptotic, and immunomodulatory role [creates a microenvironment with T and NK cells] [48] *Therapeutic Concerns to be investigated in future studies*: May be prooncogenic; risk of inherent or acquired IFN insensitivity; adverse effects (although less than with Type I IFNs) [38,50]

## Data Availability

Data sharing is not applicable to this article.
